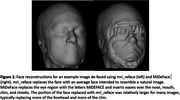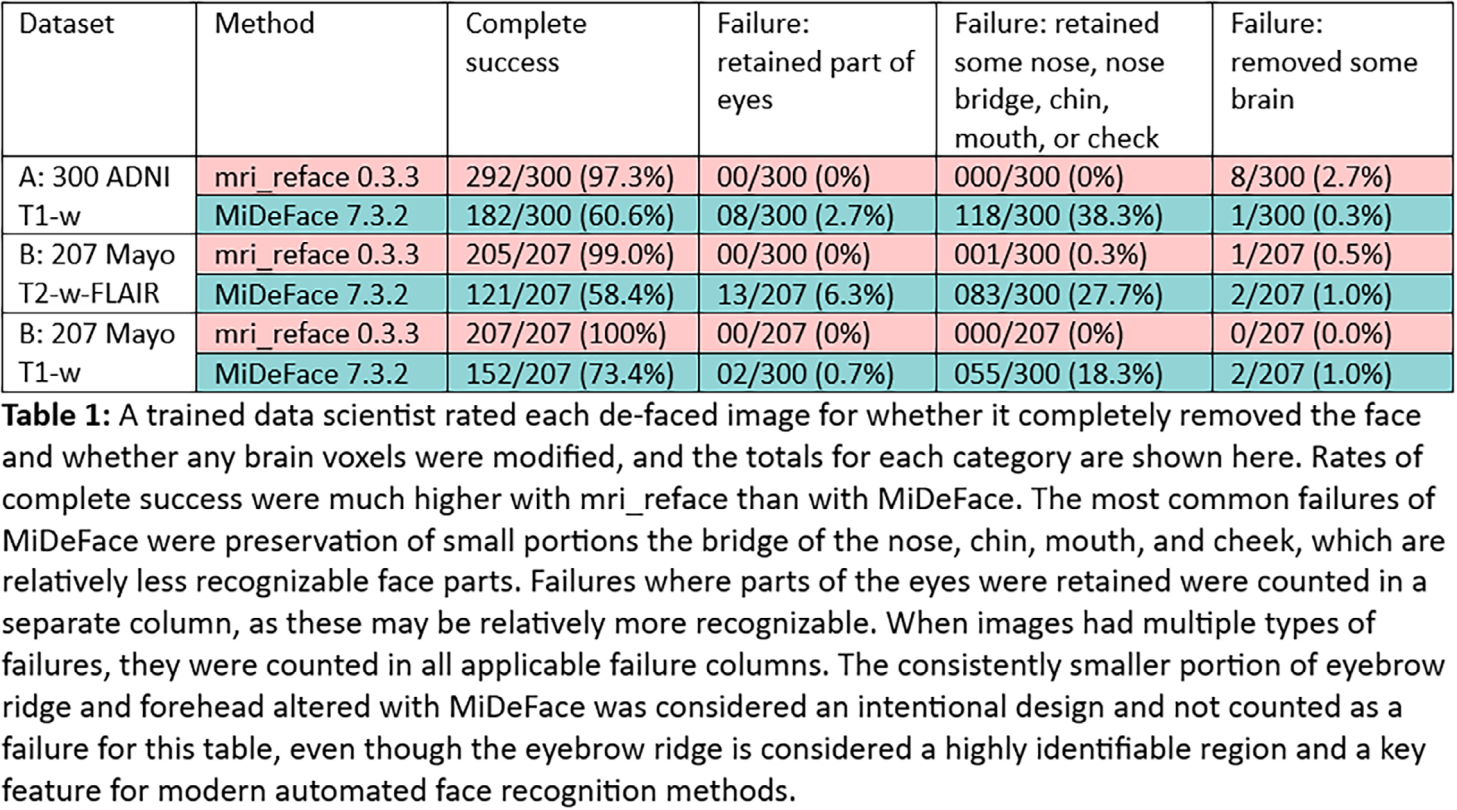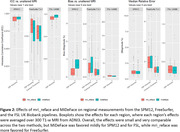# Comparing de‐facing software MiDeFace and mri_reface according to privacy protection and effects on downstream measurements

**DOI:** 10.1002/alz.091376

**Published:** 2025-01-09

**Authors:** Christopher G. Schwarz, Carl M. Prakaashana, Walter K. Kremers, Jeffrey L. Gunter, Matthew L. Senjem, Prashanthi Vemuri, Kejal Kantarci, Jonathan Graff‐Radford, David S. Knopman, Ronald C. Petersen, Clifford R. Jack, The Alzheimer's Disease Neuroimaging Initiative

**Affiliations:** ^1^ Mayo Clinic, Rochester, MN USA; ^2^ Department of Radiology, Mayo Clinic, Rochester, MN USA

## Abstract

**Background:**

Several programs are available for automatically “de‐facing” brain images. These are designed to prevent the potential use of face imagery to re‐identify research participants. Previous works have compared available software and mri_reface is frequently among the top methods. The “Minimally Invasive DeFacing of MRI images” (MiDeFace) program was more recently added to FreeSurfer, but minimal validation of MiDeFace has been published.

**Method:**

We ran mri_reface 0.3.3 and MiDeFace 7.3.2 on two datasets: A) 207 participants with face photographs and 3D T1‐weighted and 3D T2‐weighted‐FLAIR MRI from the Mayo Clinic Study of Aging (all unimpaired, Siemens); and B) 300 participants from ADNI3 with 3D T1‐weighted MRI (50 unimpaired + 50 with clinical AD, from each of 3 vendors). For dataset A, we tested the rate at which Microsoft Azure face recognition matched each participant’s photographs with the correct MRI, comparing unmodified images, those from mri_reface, and those from MiDeFace. For dataset B, we compared measured regional brain volume and cortical thickness from original vs. de‐faced images with SPM12, FreeSurfer 7.4.1, and the FSL‐UK Biobank pipeline. For both datasets, images were visually rated to assess removal of the face and retention of brain tissue.

**Result:**

Automated face recognition match rates were lower with mri_reface (17/207 8.2%) than with MiDeFace (24/207 11.6%), indicating stronger identity protection with mri_reface, but both were greatly reduced vs. unmodified images (203/207 98.1%). Example images with both programs are shown in Figure 1. From visual ratings (Table 1), rates of complete success (modification of the entire face while retaining the entire brain) were much higher with mri_reface, but the great majority of failures from MiDeFace were subtle and occurred in relatively less recognizable face regions. The effects of both programs on downstream imaging measurements (Figure 2) were small and very comparable, but MiDeFace was favored mildly for SPM12 and for FSL, while mri_reface was more favored for FreeSurfer.

**Conclusion:**

Altogether, mri_reface more reliably removed the complete face, leading to smaller rates of potential re‐identification (8.2% vs 11.6%). Effects on downstream image measurements were small and comparable with both methods.